# The development of anemia is associated to poor prognosis in NKF/KDOQI stage 3 chronic kidney disease

**DOI:** 10.1186/1471-2369-14-2

**Published:** 2013-01-07

**Authors:** José Portolés, Jose Luis Gorriz, Esther Rubio, Fernando de Alvaro, Florencio García, Vicente Alvarez-Chivas, Pedro Aranda, Alberto Martinez-Castelao

**Affiliations:** 1 Nephrology Department, Hospital Universitario Puerta de Hierro, Madrid, Spain; 2 Nephrology Department, Hospital Universitario Dr. Peset, Valencia, Spain; 3 Hospital Infanta Sofia, San Sebastian de los Reyes, Madrid, Spain; 4 Nephrology Department, Hospital Universitario Doce de Octubre, Madrid, Spain; 5 Nephrology Department, Hospital Universitario La Princesa, Madrid, Spain; 6 Nephrology Department,Hospital Carlos Haya, Malaga, Spain; 7 Nephrology Department, Hospital Universitario Bellvitge, L’Hospitalet de Llobregat, Barcelona, Spain; 8 Spanish Group for the study of Diabetic Nephropathy, GEENDIAB-SEN, Barcelona, Spain; 9 Spanish Renal Research Network, REDInRen Red 6/0016 (Instituto Salud Carlos III), Madrid, Spain; 10 Servicio de Nefrología, Hospital Universitario Puerta de Hierro, C/ Manuel de Falla 1, Majadahonda, Madrid, 28229, Spain

**Keywords:** Chronic kidney disease, Anemia, Cardiovascular risk, Non-dialysis CKD patients, Epidemiology

## Abstract

**Background:**

Anemia is a common condition in CKD that has been identified as a cardiovascular (CV) risk factor in end-stage renal disease, constituting a predictor of low survival. The aim of this study was to define the onset of anemia of renal origin and its association with the evolution of kidney disease and clinical outcomes in stage 3 CKD (CKD-3).

**Methods:**

This epidemiological, prospective, multicenter, 3-year study included 439 CKD-3 patients. The origin of nephropathy and comorbidity (Charlson score: 3.2) were recorded. The clinical characteristics of patients that developed anemia according to EBPG guidelines were compared with those that did not, followed by multivariate logistic regression, Kaplan-Meier curves and ROC curves to investigate factors associated with the development of renal anemia.

**Results:**

During the 36-month follow-up period, 50% reached CKD-4 or 5, and approximately 35% were diagnosed with anemia (85% of renal origin). The probability of developing renal anemia was 0.12, 0.20 and 0.25 at 1, 2 and 3 years, respectively. Patients that developed anemia were mainly men (72% anemic vs. 69% non-anemic). The mean age was 68 vs. 65.5 years and baseline proteinuria was 0.94 vs. 0.62 g/24h (anemic vs. non anemic, respectively). Baseline MDRD values were 36 vs. 40 mL/min and albumin 4.1 vs. 4.3 g/dL; reduction in MDRD was greater in those that developed anemia (6.8 vs. 1.6 mL/min/1.73 m^2^/3 years). These patients progressed earlier to CKD-4 or 5 (18 vs. 28 months), with a higher proportion of hospitalizations (31 vs. 16%), major CV events (16 vs. 7%), and higher mortality (10 vs. 6.6%) than those without anemia. Multivariate logistic regression indicated a significant association between baseline hemoglobin (OR=0.35; 95% CI: 0.24-0.28), glomerular filtration rate (OR=0.96; 95% CI: 0.93-0.99), female (OR=0.19; 95% CI: 0.10-0.40) and the development of renal anemia.

**Conclusions:**

Renal anemia is associated with a more rapid evolution to CKD-4, and a higher risk of CV events and hospitalization in non-dialysis-dependent CKD patients. This suggests that special attention should be paid to anemic CKD-3 patients.

## Background

Chronic kidney disease (CKD) is a highly prevalent disease frequently associated with diabetes and high cardiovascular (CV) risk. This combination results in a considerable mortality rate [1,2]. The prevalence of CKD stages 3–5 is 6.8% in Spain [3]. Anemia is common in CKD and has been linked to cardiovascular disease and mortality [2]. According to THE European Best Practice Guidelines (EBPG) published in 2004, a diagnosis of anemia is considered to be a hemoglobin concentration (Hb) of less than 11.5 g/dL in women, < 12 g/dL in men aged > 70 years, or < 13.5 g/dL in men aged ≤ 70 years [4]. Renal anemia in CKD is largely due to a deficiency in renal production of erythropoietin, usually being normocytic and normochromic, although its appearance has been also associated to iron deficiency [5], and several retrospective studies have reported a higher prevalence of anemia when inflammatory processes, malnutrition or diabetes are present in severe CKD [1,3,6,7].

The development of anemia is expected in CKD stages 3 to 5, but onset varies from one patient to another. A large cohort study performed between 1988 and 1994 reported a prevalence of renal anemia of 1% in CKD stage 3, 9% in stage 4 and over 33% in stage 5 [8]. Later studies reported higher values (5% for CKD-3 and 55% for CKD-4) [9,10].

On the other hand, CKD-3 patients may be asymptomatic, especially in the absence of anemia or other complications. Many are not referred to nephrologists and, therefore, little is known of their characteristics and evolution. Moreover, most studies on the prevalence of anemia are cross-sectional and do not establish the onset and evolution of anemia. Therefore, the primary objective of this study was to determine the time course of the clinical evolution of stage 3 CKD patients, to estimate the incidence of anemia of renal origin and to describe its characteristics.

## Methods

The NADIR-3 study (Study of non-anemic stage 3 CKD patients who develop renal anemia) is a longitudinal, prospective, multicenter study conducted in Spain. It was promoted by the Spanish Group for the Study of Diabetic Nephropathy (GEENDIAB by its Spanish initials) and the Strategic Action Plan of the Spanish Society of Nephrology. The study was performed with the approval of the reference Ethics Committee for Clinical Research of the La Paz University Hospital (Madrid), and this approval was accepted by the authorities of the other participating centers. All procedures were in compliance with the Declaration of Helsinki.

Based on the incidence of anemia and the dropout rate reported in previous studies, a minimum sample size of 427 patients was considered to estimate the anemia onset rate with a 95% precision. Recruitment of patients was performed by participant nephrologists between October 2005 and March 2006 and written consent was obtained from all participants. Clinical assessment was undertaken and blood samples of patients were obtained consecutively every 6 months for 36 months after enrollment, or until the initiation of dialysis or renal transplant (RRT). Exclusion criteria were; transfusions within three months of entry, treatment with erythropoiesis stimulating agents (ESA), prior kidney transplant, or treatment with immunosuppressive drugs. Inclusion criteria were; subjects between the ages of 18 and 78 years, with MDRD estimated glomerular filtration rates (eGFR) ≥ 30and < 60 mL/min, without anemia. Anemia was defined by the following criteria: Hb concentration below 11.5 g/dl in women, Hb below 12.0 g/dl in men aged 70 years and over, and Hb below 13.5 g/dl in men younger than 70 years of age, as stated by the EBPG guidelines [4]. The onset of anemia (anemia primary endpoint) was established as the detection of Hb concentrations below the mentioned levels at any time, and this was confirmed by a second test performed 15 days later.

Baseline clinical and biochemical data, as well as treatment information was obtained during the first visit and follow-up data were obtained every six months until completion of a 36–month follow-up period. All data were included in an electronic CRF. Recorded demographic, clinical and biochemical data included age; gender; etiology of renal disease; time from CKD-3 diagnosis; smoking habits; weight; height; body mass index (BMI); medications; blood pressure (BP); serum values of glucose, lipid profile, total plasmatic proteins, albumin, urea, uric acid, creatinine, potassium, bicarbonate, calcium, phosphate, parathyroid hormone (PTH), alkaline phosphatase, C-reactive protein (CRP), homocysteine, ferritin, transferrin saturation index, folic acid, and vitamin B12; CBC; 24-hour diuresis; urine urea and proteinuria. Glomerular filtration was estimated using the MDRD-4 formula, based on non-standardized serum creatinine values in each center.

The development of anemia was continuously recorded, including an exhaustive clinical study to identify the cause of its appearance as suggested by clinical guidelines [4]. Anemia of renal origin was established once other possible causes of anemia were excluded (episodes of bleeding, surgery, chronic inflammation, myelodysplasic syndrome, tumors, folic acid or vitamin B12 deficit, other minor causes). Onset of anemia was analyzed in fixed annual periods and events (hospital admissions, death) were considered only after the onset of anemia in order to analyze the possible association. This allowed for the establishment of a logical link provided by timing.

Patients were instructed to contact their nephrologist if any Hb value under the aforementioned anemia limits was present in any blood test performed during the study period, including tests ordered for other reasons or by other physicians. Likewise, a note to the primary care physician was included in every clinical report handed to the patients. Supervening events as hospitalization, the initiation of RRT, major CV events, or death were also recorded throughout the study. As a secondary objective, we included a descriptive analysis of these events and the factors related with their appearance. Comorbidities at the time of inclusion were calculated by the Charlson comorbidity index [11,12]. Diabetes and dyslipidemia were diagnosed according to the American Diabetes Association and the National Cholesterol Education Program criteria.

Data were analyzed using the SPSS 12.0 statistical package (SPSS Inc., Chicago, Illinois, USA). Categorical variables are presented as the number of observations (n) and percentages. Continuous variables are expressed as mean and standard deviation (SD) unless otherwise indicated. Comparisons between categorical variables were analyzed with the *χ*2 test or the Z-test. Continuous variables were compared using parametric or non-parametric tests as appropriate (*t*-test or one-way ANOVA if parametric, Wilcoxon signed rank sum test or ANOVA if non-parametric). ROC curves were performed to analyze the specificity and sensitivity of the development of anemia as a prognostic factor in CKD. Time to onset of anemia (primary event) was calculated using Kaplan-Meier curves. Spearman’s rank-order correlation coefficient and multivariate logistic regression analysis were used to examine the relationship between baseline status (renal disease, Hb, comorbidities) and the development of anemia. Survival analysis was adjusted for competing risks (death, renal transplant and patient drop out prior to the development of anemia) to avoid overestimation of risk. For all analyses, a P-value < 0.05 was deemed statistically significant.

## Results

**Figure 1:**
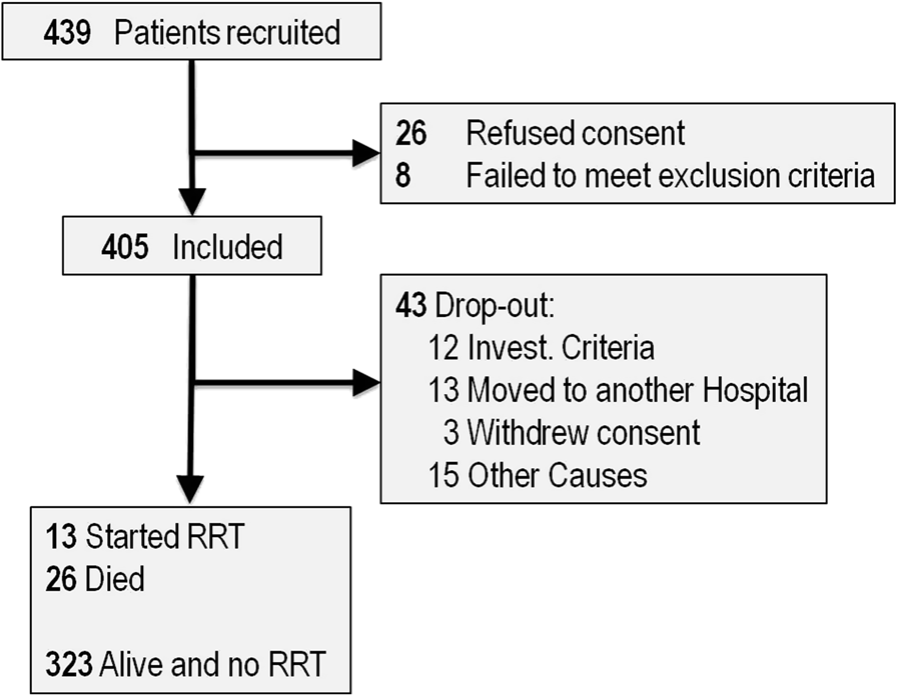
Flowchart of subjects included in the study.

Twenty-seven nephrology departments in Spanish hospitals participated in the NADIR-3 study. Four hundred and thirty nine patients meeting inclusion criteria were enrolled. Figure 1 shows the patient flowchart.

### Baseline characteristics of participants

Table 1 summarizes the demographic and laboratory data of the patients included. Etiology of renal disease was: vascular nephropathy (28.4%), disease of unknown etiology (18.0%), diabetic nephropathy (17.0%), glomerular (12.3%), interstitial (10.4%), or polycystic disease (7.2%) and disease of other origin (6.7%). Most patients (78.0%) were referred to nephrologists or had been diagnosed in the 5 years prior to recruitment. Estimated glomerular filtration rate was calculated using the abbreviated MDRD method (eGFR), and baseline values were 39.1 ± 9.1 mL/min. Hemoglobin mean value (Hb) was 14.3 ± 1.3 g/dl (range 11.6-20.4) per inclusion criteria, and was higher in men than in women (14.7 g/dl [range 12.0-20.4] vs. 13.3 g/dl [range 11.6-16.4], respectively). In men ≥ 70 years, mean baseline Hb was 14.3 g/dl (range 12.0-18.3), which was lower than in younger men (14.9 g/dl [range 13.5-20.4]). Baseline eGFR and Hb values did not correlate in women and in men ≥ 70 years, while there was a significant correlation in men younger than 70 years of age (p< 0.01).

**Table 1:** CKD-3 patient baseline characteristics classified by the development or not of renal anemia

		**Total**	**Non-anemic patients n = 242**	**Anemic patients n = 102**	**Statistical significance**
**Gender**	Female [n (%)]	122 (30.1%)	93 (30.7%)	29 (28.4%)	0.976
**Age (years)**	Median (range)	67 (22–78)	68 (22–78)	65 (24–78)	0.115
**Time from CKD diagnosis (years)**	Mean (SD)	4.1 (4.8)	3.9 (4.8)	4.7 (4.9)	0.119
**Smoking [n, (%)]**	Smokers	41 (10.1%)	28 (9.2%)	13 (12.7%)	0.427
	Ex-smokers	158 (39.0%)	116 (38.3%)	42 (41.2%)	
**BMI (Kg/m**^**2**^**)**	Mean (SD)	28.0 (4.1)	27.9 (3.9)	28.5 (4.6)	0.260
**Blood pressure (mmHg)**	Systolic [mean (SD)]	134.3 (16.0)	133.8 (15.7)	135.7 (16.8)	0.302
	Diastolic [mean (SD)]	76.3 (10.2)	75.6 (9.8)	78.5 (10.8)	0.013
**MDRD-eGFR (mL/min)**	Mean (SD)	39.1 (9.1)	40.1 (9.3)	35.9 (7.9)	0.000
**uProtein (g/24 h)**	Mean (SD)	0.7 (1.1)	0.6 (0.9)	1.0 (1.4)	0.011
**Serum Albumin (g/dL)**	Mean (SD)	4.3 (0.4)	4.1 (0.4)	4.3 (0.3)	<0.001
**Corrected Calcium (mEq/L)**	Mean (SD)	9.4 (0.5)	9.4 (0.5)	9.5 (0.43)	0.005
**Phosphate (mg/dl)**	Mean (SD)	3.4 (0.7)	3.4 (0.7)	3.7 (0.6)	0.000
**PTH (pg/ml)**	Mean (SD)	95.9 (63.8)	91.9 (50.3)	107.4 (92.0)	0.181
**CRP (mg/dl)**	Mean (SD)	2.1 (3.0)	2.2 (3.0)	1.7 (2.9)	0.143
**Ferritin (ng/dl)**	Mean (SD)	131.8 (0.6)	128.9 (93.4)	139.6 (104.9)	0.365
**Transferrin saturation index (%)**	Mean (SD)	30.2 (31.1)	31.4 (35.9)	26.9 (8.0)	0.278
**Folic acid (ng/mL)**	Mean (SD)	8.7 (3.6)	8.6 (3.6)	9.0 (3.7)	0.434
**Vitamin B12 (pg/mL)**	Mean (SD)	501.6(196.2)	495.1 (197.5)	518.9 (193.1)	0.347
**Hb (g/dl)**	Mean (SD)	14.2 (1.7)	14.0 (1.3)	13.7 (2.5)	0.000
**Medications**	Antihypertensives [n (%)]	378 (93.3%)	278 (91.7%)	100 (98.0%)	0.028
	Anticoagulant / antiplatetet [n (%)]	31 (7.7%)	27 (8.9%)	4 (3.9%)	0.101
	Glucose lowering agents [n (%)]	117 (28.9%)	88 (29.0%)	29 (28.4%)	0.906
	Lipid lowering agents [n (%)]	242 (59.8%)	180 (59.4%)	62 (60.8%)	0.806
	Mineral bone treatment [n (%)]	41 (10.1%)	30 (9.9%)	11 (10.8%)	0.798
	Others [n (%)]	154 (39.5%)	120 (41.2%)	34 (34.3%)	0.225

Data are mean (standard deviation) or median and range, as indicated. n = number of patients. BMI: Body Mass Index. MDRD-eGFR: estimated glomerular filtration rate calculated with the abbreviated MDRD equation. CKD: Chronic kidney disease. PTH: parathyroid hormone. CRP: C-reactive protein. Hb: Hemoglobin.

Most patients (92.8%) presented with hypertension and approximately one third (32.8%) were diabetic at the time of inclusion. Charlson Comorbidity Index (CCI) at baseline was 3.22 ± 1.58 (range 2–12; median 3.00). None of the patients presented with a history of bleeding events.

### Evolution of renal function and associated disease conditions

**Figure 2:**
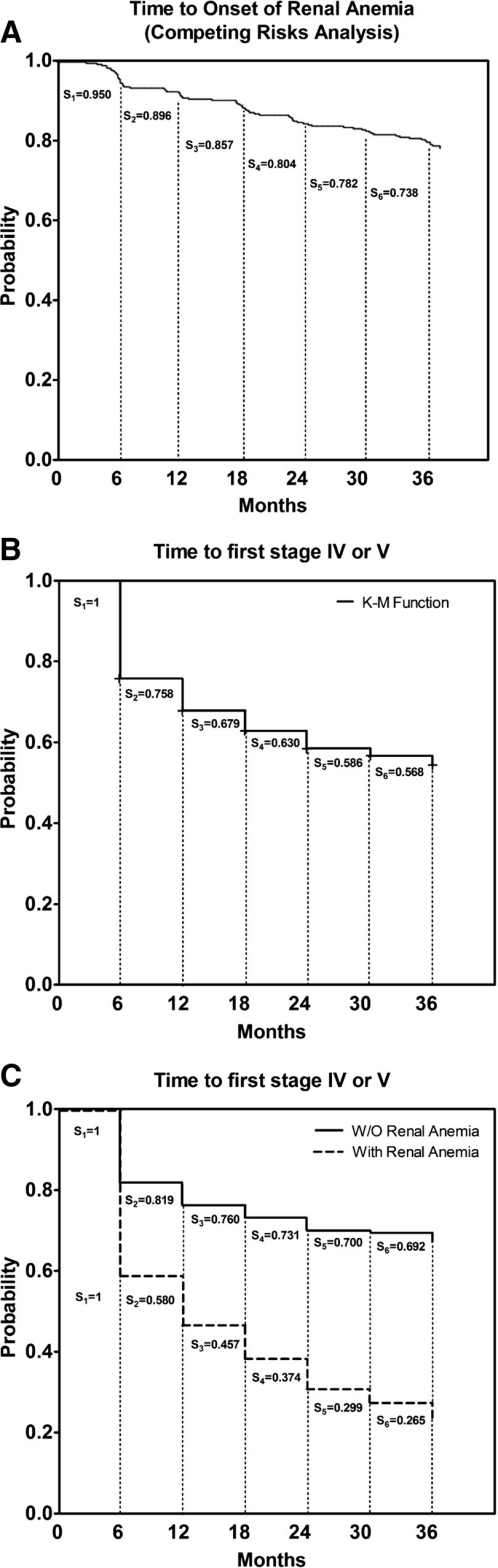
**Survival distribution function according to: A. Time to development of anemia (competing risk model with death and RRT). B**. Evolution to CKD-4 or CKD-5 for all the CKD-3 patients included in the study (Kaplan-Meyer). **C**. Evolution to CKD-4 or -5 for patients that developed renal anemia (Yes) and the non-anemic patients (No) (Kaplan-Meyer). See text for details.

The eGFR decline rate was 1.1 mL/min per year and the mean value at the end of study was 36.0 ± 12.3 mL/min vs. 39.1 ± 9.1 mL/min at the time of inclusion. As per inclusion criteria, all the patients presented CKD-3 at baseline, and 173 patients were diagnosed CKD stage 4 or 5 during the 36 month duration of the study. Kaplan-Meier analysis showed an estimated survival time of 25.4 ± 0.7 months until stage 4–5 (Figure 2B). Proteinuria was also significantly reduced at the end of the follow-up period (0.69 ± 1.00 g/24h vs. 0.52 ± 0.80 g/24 h, p< 0.05). The proportion of patients meeting the criterion of relevant proteinuria (> 1.0 g/24 h) decreased from 20.4% to 15.1%, but the difference was not significant (p=0.114). No changes in mean blood pressure (systolic, diastolic and pulse) were observed, and the distribution of patients according to blood pressure targets did not vary (data not shown). Similarly, the proportion of patients using anti-hypertensive medications did not change during the study period (95.4% vs. 96.3% at 36 months), although the average number of anti-hypertensive medications used increased in the same period (2.2 to 2.4 per patient). No variation in the proportion of patients on treatment with angiotensin-converting enzyme inhibitors was observed, while the proportion of patients using angiotensin II receptor antagonists slightly increased (54% *vs.* 48%).

None of the patients displayed malnutrition, defined as BMI < 20 and albumin < 3.5 g/dl, at any time point. Mean BMI did not change significantly (data not shown), and there was a small but significant decrease of serum albumin concentration from 4.3 ± 0.4 to 4.2 ± 0.4 g/dl (p< 0.005).

Twenty-two patients developed *de novo* diabetes during the follow-up period. Although the mean concentration of serum glucose and glycosylated Hb values did not change with time, there was a reduction in the proportion of patients with glycosylated Hb values below 7.5% (79.8% compared to 69.7% at baseline; p< 0.001).

### Time course of hemoglobin concentration changes and onset of renal anemia

At the end of the 3-year follow-up period, 120 patients developed anemia. Anemia appeared after hemorrhage in 8% of these patients, after surgery in 3%, and after a diagnosis of neoplasia in 1%; less than 1% were diagnosed with megaloblastic anemia. Anemia of other origin was reported in 3%. The rest (85%, n=102) were diagnosed with renal anemia according to the established criteria. Onset of renal anemia varied with time: 34 patients were diagnosed at 6 months; 14 at 12 months; 18 at 18 months; 14 at 24 months; 10 at 30 months and 12 at 36 months. The mean time from inclusion until diagnosis of renal anemia, in those patients who developed it, was 16.8 ± 10.2 months (range 2.9-35.9). Patients with renal anemia who presented with Hb below 11 g/dl were treated with erythropoietic stimulating agents (27.3% of anemic patient at 36 months). Use of iron therapy increased progressively to 21% at the end of follow-up. Only 13 patients were on ESA treatment for more than one year. ESA treatment did not have any effect on patient outcome (data not shown) possibly due to the low percentage of patients receiving treatment and for short times. Although treatment of anemia and analysis of the degree of target fulfillment were not among our objectives, it should be emphasized that there is a wide margin between the definition of anemia and the level of Hb where treatment with ESAs should be started. Therefore, the low percentage of patients treated with ESAs cannot be interpreted as “therapeutic nihilism*”.*

Kaplan-Meier analysis was performed to estimate the onset of renal anemia. The incidence rate was about 9 cases per 1000 patient-months and the estimated mean survival time until anemia onset (i. e., time in which the probability of developing anemia reaches 50% for all patients) was 34.1 ± 0.7 months, 95% CI [32.7-35.4]. Since this event did not reach 50% at the time of study closure, the time median could not be estimated. A survival analysis plot adjusted for competing risks (exitus, renal transplant and patient drop out before anemia development) was used to avoid overestimation of the incidence of anemia (Figure 2A). The estimated annual rate of onset of renal anemia was 0.11 in the first year, 0.20 in the second year and 0.26 in the third year,

### Clinical factors associated with the development of renal anemia and the evolution of anemic and non-anemic patients

Table 1 shows the characteristics of patients that developed renal anemia during the 36-month follow-up period (anemic patients) and those that did not (non-anemic patients). Baseline clinical and analytical data were compared. As can be observed, baseline values of proteinuria and serum albumin were significantly different between patients with and without anemia. Similarly, baseline MDRD-eGFR was significantly lower in anemic patients than in non-anemic patients. Table 2 shows the main clinical events and outcomes in patients classified according to the onset of anemia.

**Table 2:** **Main clinical evolution parameters in patients who developed renal anemia *vs. *non-anemic patients**

	**Non-anemic patients**	**Anemic patients**	**p-value**
**Mortality**	6.6%	10.3%	p<0.005
**Annual hospitalization rate**	16.1%	31.4%	p<0.001
**Major CV events**	7.2%	16.4%	p<0.01
**Δ MDRD (mL/min/3 years)**	-1.6	-6.8	p<0.001
**Reached CKD 4-5**	32.8%	74.3 %	p<0.001

Δ MDRD: difference in estimated glomerular filtration rate calculated with the abbreviated MDRD equation. CDK: Chronic kidney disease.

**Figure 3:**
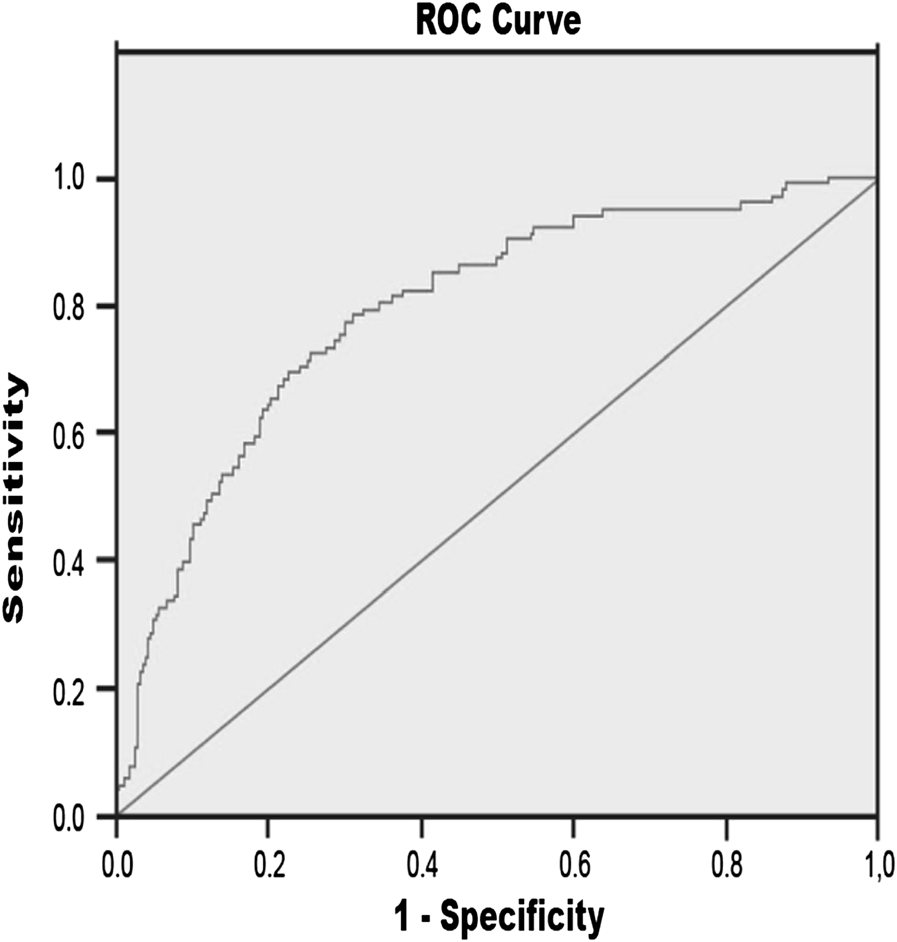
Receiver operator characteristic (ROC) curve showing the sensitivity and specificity of the model incorporating eGFR, HB and gender to predict development of anaemia within the 3-year follow-up period.

Performance of multivariate logistic regression analysis to identify factors related to the development of anemia led to a best model that included baseline eGFR, baseline Hb value and gender (Table 3). The risk of anemia increased 2.88-fold with each reduction of 1 g/dl in the initial Hb concentration increased 1.04-fold per each reduction of 1 mL/min in the eGFR-MDRD value was 5.05 times higher for male subjects (OR=0.198; p< 0.001) (Table 3). Baseline proteinuria and comorbidities did not enhance the predictive value of this model. ROC curves were generated considering this 3-variable model and specificity of 92.3%, sensitivity of 34.7% and area under the curve 0.792 (0.741-0.843) were obtained (Figure 3). The low sensitivity value indicates that the mathematical model appropriately discriminates non-anemic patients only.

**Table 3:** Risk factors for renal anemia development. Odds ratio (OR) values for multivariate backward step logistic regression

	**B**	**P value**	**OR**	**95% CI**
**Basal Hb value (per ↑ 1 g/dl)**	-1.06	0.000	0.347	[0.253 - 0.475]
**Basal eGFR MDRD (per ↑ 1 mL/min)**	-0.04	0.012	0.961	[0.931 - 0.99]
**Female vs. Male**	-1.62	0.000	0.198	[0.099 - 0.399]

MDRD-eGFR: estimated glomerular filtration rate calculated with the abbreviated MDRD equation. Hb: Hemoglobin.

The incidence of hospitalizations during the follow-up was 16.1% in non-anemic patients, the median number of hospitalizations per patient being 1 (range 1–2), and the median number of days in the hospital being 5 (range 1–36) . The main reason for hospital admission in non-anemic patients was surgery, followed by infection, peripheral vascular disease, angina, and other CV events. The frequency of hospital admissions was significantly higher (31.4% vs. 16.1%; p< 0.001) in anemic patients, the median number of hospitalizations per patient being one (maximum 3) and the median number of days in the hospital 10 (range 2–36). The most common reason in these patients was congestive cardiac failure, followed by infection, surgery, peripheral vascular disease, and other cardiovascular events. In the anemic group, the incidence of major CV events after three years of follow-up was significantly higher (Table 2). These results support the idea that development of anemia CKD-3 is associated to a more rapid evolution of clinical condition.

As stated above, 173 patients reached stages 4 or 5 during the study. Of these, 75 developed renal anemia (74.3% of all anemic patients) and 78 did not (32.2% of all non-anemic patients) (Table 2). Survival analysis showed significant differences between both groups (Figure 2C). The average estimated time to reach CKD-4 or CKD-5 was 28.2 ± 0.8 months for non-anemic patients and 18.0 ± 1.3 months for anemic patients, and the difference between these values was significant (p< 0.001). Quicker progression of renal disease was found between groups when the decrease of eGFR was compared (-6.8 ml/min/36 months vs. -1.6 mL/min/36 months; p< 0.001). Despite the greater reduction in eGFR found in anemic patients, the decrease in proteinuria over the follow-up period was slightly lower in anemic patients (20.9% vs. 29.1%, p=0.240). The quicker decrease of eGFR and the shorter time to reach CKD-4 or -5 suggested that development of renal anemia could be a surrogate marker indicating faster progression of kidney disease.

## Discussion

Anemia is a well-known complication of CKD but there is controversial information about its onset, morbidity and relevance as a prognostic factor in stage 3 CKD. The present longitudinal study included more than 400 CKD-3 patients that were non-anemic at baseline. Diagnosis of anemia in this CKD-3 group increased with time, approaching 35% at 36 months. Previous epidemiological studies established a prevalence of anemia of around 5% in CKD-3 and 45% in CKD-4 stages [8,10,13]. However, most of these studies included patients in different CKD stages who were often subject to dialysis or transplant, or were single-center studies with a low number of participants. The prevalence of anemia at the end of the follow-up period in our cohort was only slightly lower than that previously reported in transversal studies, but comparison is difficult due to the significant differences in study design and patient populations. A previous preliminary interim analysis of this study showed that 12.4% of patients developed anemia during the first year of follow-up [14]. In this final analysis, prevalence increased by about 10% per year in a real setting of follow-up in nephrology services, and was accompanied by renal function decline of 1.1 mL/min per year. To our knowledge, this is the first prospective estimation of the rate of development of anemia in CKD 3 patients.

CKD-3 patients that developed anemia in our cohort had a significant increase of risk of hospital admission, CV events and mortality when compared to non-anemic CKD-3 patients. We also observed that a quicker decrease in the eGFR and increased proteinuria were associated with the development of anemia, and this condition has been associated with increased morbidity and mortality in CKD patients [1,2,15,16]. Two studies performed with young CKD-3 to CKD-5 patients showed that anemia is associated with diminished quality of life, increased frequency of hospitalization and higher risk of mortality[15,17], while another did not find any association between anemia and increased hospitalization [18]. These controversial results may be due to different patient characteristics, comorbidity, CKD stages of the cohort, sample sizes or anemia definitions. The lack of consistency in the results between these studies emphasizes the importance of studying homogeneous groups of patients and, in particular, the need to describe *“predialysis CKD patients”* separately from those that receive renal replacement therapy. This has been one of the objectives of the present work. Our results indicate a poorer prognosis for patients who develop anemia. There is a possibility that anemia, even anemia of renal origin, acts as a surrogate marker for other conditions associated to increased risk of hospitalization, that is, other underlying diseases or chronic inflammation, that have been associated with anemia in CKD patients [19,20]. This possibility should be investigated in further studies. In the present study, the most common cause for hospitalization in anemic patients was congestive heart failure, which could be due to the additive effect of anemia on the cardiac condition caused by hypertension, which was present in almost all the participants [21] .

A recent prospective study including more than 600 anemic and non-anemic CKD patients classified them in four categories (non-anemic, mild anemic with corrected anemia, persistent mild anemic or progression to severe anemia), and the last two groups showed higher risk for progression to end-stage renal disease or death. [22]

Previous studies have also reported the association between anemia and CKD stage [2,7-10,13,23-26]. Similarly, our results showed decreased kidney function in anemic patients compared to non-anemic participants, as revealed by the faster decrease of GFR and, consequently, the shorter time to reach stages CKD-4 or CKD-5. It not possible to determine which is the first event, anemia or CKD progression, but the present results suggest that anemia may be a potential marker for faster progression of CKD, and for the probability of hospitalization and major CV events. In the present series, those patients that did not develop anemia showed better clinical evolution, associated with a slower progression of CKD as well as lower rates of mortality and relevant clinical complications.

Several previous reports indicated the association of baseline eGFR, proteinuria, greater age and male gender with a higher prevalence of anemia [13,23,25,26]. We have found that patients that developed anemia had significantly lower baseline eGFR and higher proportion of significant proteinuria (>1 g/24h) [10,14]. However, proteinuria did not have prognostic value in the multivariate model including initial eGFR, Hb, and gender. This suggests a need for closer follow-up of non-anemic CKD-3 patients with high proteinuria and progression of CKD in order to detect renal anemia onset early and eventually adjust treatment.

Although the number of participants in our study was sufficient, some limitations should be commented: Firstly, the observational design does not allow for the establishment of causality between the onset of anemia and poor outcomes; Secondly, selection bias in our cohort is possible, since inclusion depended on patient acceptance; and thirdly, the study lacked a consensus protocol for starting dialysis, so we could not consider it as an event. Finally, although data on the onset of anemia was collected at a precise time point, other data were collected at 6-month intervals, and the results obtained from time-dependent analysis should be interpreted cautiously. However, the resulting cohort was in accordance with the sample-size estimation and presented great homogeneity, probably being the largest group in which the characteristics of CKD-3 patients have been studied.

## Conclusions

In summary, we have estimated the timing and rate for the onset of anemia in CKD-3 patients in a specific 3-year prospective study using a competing risk model. The probability of developing anemia was higher for men, those of older age, lower baseline eGFR and higher proteinuria levels. Only some patients who developed anemia reached the Hb level necessary to be eligible for treatment with ESAs. Anemia was also associated with a rapid decline of renal function, increased risk of hospitalization and increased mortality. Those CKD-3 patients who did not develop anemia presented relatively good prognoses and better outcomes. The present overall results suggest that the onset of anemia in CKD stage 3 distinguishes two types of patients with different clinical evolutions, which may help to establish different health strategies for these patients.

## Abbreviations

CKD: Chronic kidney disease; CV: Cardiovascular; ESA: Erythropoiesis-stimulating agents; ESRD: End-stage renal disease; eGFR: Estimated glomerular filtration rate; GFR: Glomerular filtration rate; Hb: Hemoglobin levels; MDRD: Modification of diet in renal disease; RRT: Renal replacement therapy

## Competing interests

The authors’ state no conflict of interest apart from the grant support from Amgen S.A. indicated above.

## Authors’ contributions

JP, JLG, FA and AMC conceived the study and participated in its design. These authors were the scientific committee coordinators and principal investigators in each of their hospitals, coordinating work in each group. ER contributed in data collection and analysis and participated in article draft writing and revision. FG, VAC and PA contributed in data collection and analysis and participated in advanced draft revision. All other contributors participated in data collection, preliminary discussion and final analysis. All contributors attended two early stage meetings, another halfway through the study and one final meeting. All authors read and approved the final manuscript.

## Authors’ information

**This study has been conducted by the above-signed authors and other members of the NADIR-3 Study Group-GEENDIAB-SEN** (in alphabetical order): Arteaga J, H. de Navarra (Pamplona); Borrego J., Complejo Hospitalario de Jaén (Jaen); Bustamante J, H. Clínico de Valladolid (Valladolid); Calero F, Fundació Puigvert (Barcelona); Cases A, H. Clínic (Barcelona); Conde J, H. de Toledo (Toledo); Fernández-Vega F and Camino, H. Central de Asturias (Oviedo); Íñigo P, H. Clínico Universitario Lozano Blesa (Zaragoza); Gascó J, H. Son Llatzer (Palma De Mallorca); Granda M, H. Virgen Blanca (León); Graña JM, H. de La Rivera (Valencia); Gruss E, Fundación H. de Alcorcón (Madrid); Lorenzo D, H. Juan Canalejo (A Coruña); Llópez-Carratalá MR, H. Puerta de Hierro (Madrid); Martínez-Castelao A, H. de Bellvitge (Barcelona); Martínez-Fernández I, H. de Galdakao (Vizcaya); Dr. Nieto J, H. Gral. de Ciudad Real (Ciudad Real); Otero A, Complejo H. de Ourense (Ourense); Robles NR, H. Infanta Cristina (Badajoz); Sánchez-Casajús A, Complejo H. San Millán-San Pedro (Logroño); Sanjuán A, H. Universitario Miguel Servet (Zaragoza); Pérez-Fontán M, H. Juan Canalejo (A Coruña); Sánchez-Palencia R, H. Virgen de La Macarena (Sevilla); Sanz de Castro S, H. Marqués de Valdecilla (Santander).

H. la Princesa, H. Puerta de Hierro, H. Marqués de Valdecilla, Fundación H. Alcorcon and Fundacion Puigvert are in RedInRen (Red de Investigación Renal –Renal Research Network).

## Pre-publication history

The pre-publication history for this paper can be accessed here:

http://www.biomedcentral.com/1471-2369/14/2/prepub
